# miR-15a and miR-20b sensitize hepatocellular carcinoma cells to sorafenib through repressing CDC37L1 and consequent PPIA downregulation

**DOI:** 10.1038/s41420-022-01094-2

**Published:** 2022-06-27

**Authors:** Li Li, Shijun Yu, Jingde Chen, Ming Quan, Yong Gao, Yandong Li

**Affiliations:** 1 Department of Oncology, Shanghai East Hospital, Tongji University School of Medicine, Shanghai, 200120 China; 2 Research Center for Translational Medicine, Shanghai East Hospital, Tongji University School of Medicine, Shanghai, 200120 China

**Keywords:** Cell death, Cancer therapeutic resistance

## Abstract

Sorafenib is a classical targeted drug for the treatment of advanced hepatocellular carcinoma (HCC), but intrinsic resistance severely limited its therapeutic effects. In the present study, we aimed to identify crucial genes in HCC cells that affect sorafenib resistance by a CRISPR/Cas9 genome-scale screening. The results indicated that the deficiency of miR-15a and miR-20b contributed to sorafenib resistance, whereas exogenous expression of miR-15a and miR-20b enhanced sorafenib sensitivity of HCC cells by cell viability, colony formation, and flow cytometry analyses. Further analyses revealed that cell division cycle 37 like 1 (CDC37L1) as a common target of miR-15a and 20b, was negatively regulated by the two miRNAs and could enhance sorafenib resistance of HCC cells in vitro and in vivo. Mechanistically, CDC37L1, as a cochaperone, effectively increased the expression of peptidylprolyl isomerase A (PPIA) through strengthening the binding between heat shock protein 90 (HSP90) and PPIA. The results from immunohistochemical staining of a HCC tissue microarray revealed a positive association between CDC37L1 and PPIA expression, and high expression of CDC37L1 and PPIA predicted worse prognosis of HCC patients after sorafenib therapy. Taken together, our findings reveal crucial roles of miR-15a, miR-20b, CDC37L1, and PPIA in sorafenib response of HCC cells. These factors may serve as therapeutic targets and predict prognosis for HCC treated with sorafenib.

## Introduction

Hepatocellular carcinoma (HCC) is one of most common malignancies with the third cancer-related mortality worldwide, seriously threatening to human health [1]. Due to the insidious onset, rapid progress and low early diagnosis rate, most patients with HCC are diagnosed already at the advanced stage, resulting in poor therapeutic effects of surgical resection or liver transplantation [2]. Sorafenib, an oral multi-kinase inhibitor of Raf, VEGFR2, PDGFR, FLT3, and KIT receptor tyrosine kinase, has been approved by the American Food and Drug Administration (FDA) since 2007 and it is also the first standard clinical drug for the treatment of unresectable advanced HCC patients [3]. However, sorafenib only benefits about 30% of patients, high rate of drug resistance is regarded as a major obstacle limiting its therapeutic effects [4, 5]. Therefore, exploring the underlying mechanisms of sorafenib resistance and identifying effective biomarkers to predict the response of patients to sorafenib treatment are of great significance for improving the applications of sorafenib.

Previous studies have reported many factors and signaling pathways involved in sorafenib resistance of HCC cells, including autophagy-related gene dysregulation, EGFR signaling activation, and expression alteration of several microRNAs (miRNAs) [6, 7]. For example, elevated nuclear protein 1 (NUPR1), vaccinia-related kinase 2 (VRK2) and reduced let-7, miR-142-3p, miR-34a, and miR-486-3p expression are observed in sorafenib-resistant HCC and these genes are found to correlate with autophagy or apoptosis [8–13]. The upregulation of miR-23a-3p and phosphoseryl-tRNA kinase (PSTK) and the downregulation of Yes1 associated transcriptional regulator (YAP) blunt HCC sorafenib efficacy via inhibiting ferroptosis [14–16]. In the modifications of sorafenib resistance, miRNAs are important regulators involved in various cellular processes through their targeted genes [6, 17]. However, most of miRNAs were identified based on acquired resistance, which means the expression alteration of these miRNAs occurred after treatment of sorafenib. The intrinsic resistances caused by miRNAs have not been fully elucidated to date.

With the development of CRISPR/Cas9 gene-editing technology, several CRISPR pooled libraries have been established and used for high-throughput genetic screening for those genes involved in tumor growth, metastasis or drug resistance [18–20]. Accumulating studies have identified multiple potential drivers of sorafenib resistance in HCC, such as phosphoglycerate dehydrogenase (PHGDH), shugoshin 1 (SGOL1), and metaxin 1 (MTX1) [19, 21, 22]. Besides, through this screening method, a recent study also identifies Kelch-like ECH-associated protein 1 (KEAP1) as a sorafenib, regorafenib, and lenvatinib sensitivity gene in HCC [23]. To extend the knowledge of sorafenib resistance, we used the genome-wide CRISPR-Cas9 knockout screen to identify potential regulatory factors in sorafenib resistance, and a series of candidates were successfully screened out. Among of them, we focused on two miRNAs, miR-15a, and miR-20b, inhibition of which showed relative strong cell viability upon sorafenib treatment. Subsequently, Cell division cycle 37 like 1 (CDC37L1) was identified as a common target of miR-15a and miR-20b. CDC37L1 may act as a cochaperone to enhance the interaction between heat shock protein 90 (HSP90) and peptidylprolyl isomerase A (PPIA), thereby increasing stability of PPIA and finally contributing to sorafenib resistance of HCC.

## Results

### CRISPR/Cas9 loss-of-function screen identified miR-15a and miR-20b as critical genes involved in HCC sorafenib resistance

**Fig. 1:**
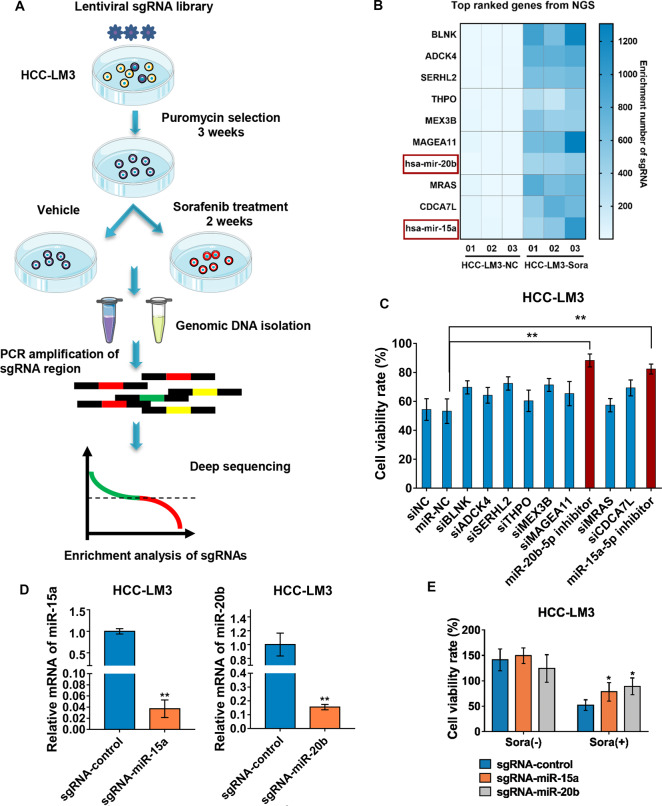
Genome-Scale CRISPR/Cas9 Screen identified miR-15a and miR-20b involved in sorafenib response. **A** Schematic of screening for sorafenib resistance-associated genes in HCC-LM3 cells. **B** Heat map displayed the rank of sgRNA abundance according to enrichment levels in sorafenib-resistant HCC-LM3 cells. **C** CCK8 assays were performed in HCC-LM3 cells with silencing of targeting gene as indicated, respectively (one-way ANOVA). **D** The expression analysis of miR-15a and miR-20b by qRT-PCR (unpaired *t* test, two-tailed). **E** The ablation of miR-15a and miR-20b improved cell viability under sorafenib (7.5 μM) treatment (one-way ANOVA). *p < 0.05, **p < 0.01.

To identify key genes involved in sorafenib resistance, we performed a genome-wide CRISPR/Cas9 knockout (GeCKO v2) library screen in HCC-LM3 cell line. The screening procedures were shown in Fig. 1A. The results showed that 108 sgRNAs were upregulated more than 2-fold in the sorafenib treatment group. Then these positive hits were ranked according to the enrichment of sgRNAs, and the targeting genes of top 10 sgRNAs included BLNK, ADCK4, SERHL2, THPO, MEX3B, MAGEA11, hsa-miR-20b, MRAS, CDCA7L, hsa-miR-15a (Fig. 1B). To validate the screening results, we knocked down the 10 genes’ expression respectively through transfecting specifically siRNAs or miRNAs-targeted inhibitors into HCC-LM3 cells, and then accessed the cell viability rate after treatment with sorafenib for 48 h. Among the 10 genes, silencing of miR-15a or miR-20b effectively increased HCC cell viability in the presence of sorafenib, enhancing resistance of HCC cells (Fig. 1C), consistent with the sequencing data that the hits of enriched sgRNAs targeting miR-15a and miR-20b in sorafenib-resistant cells increased by 38 times and 59 times respectively. Meanwhile, CRISPR/Cas9-mediated deletion of miR-15a or miR-20b in HCC-LM3 cells was established (Fig. 1D). Cell viability assay results showed deletion of miR-15a or miR-20b significantly increased cell viability rate (Fig. 1E) and formed more and bigger colonies than control cells in the presence of sorafenib (Supplementary Fig. S1). These results indicated that miR-15a and miR-20b may play an essential role in HCC during sorafenib treatment.

### Overexpression of miR-15a and miR-20b conferred sorafenib sensitivity of HCC cells in vitro and vivo

**Fig. 2:**
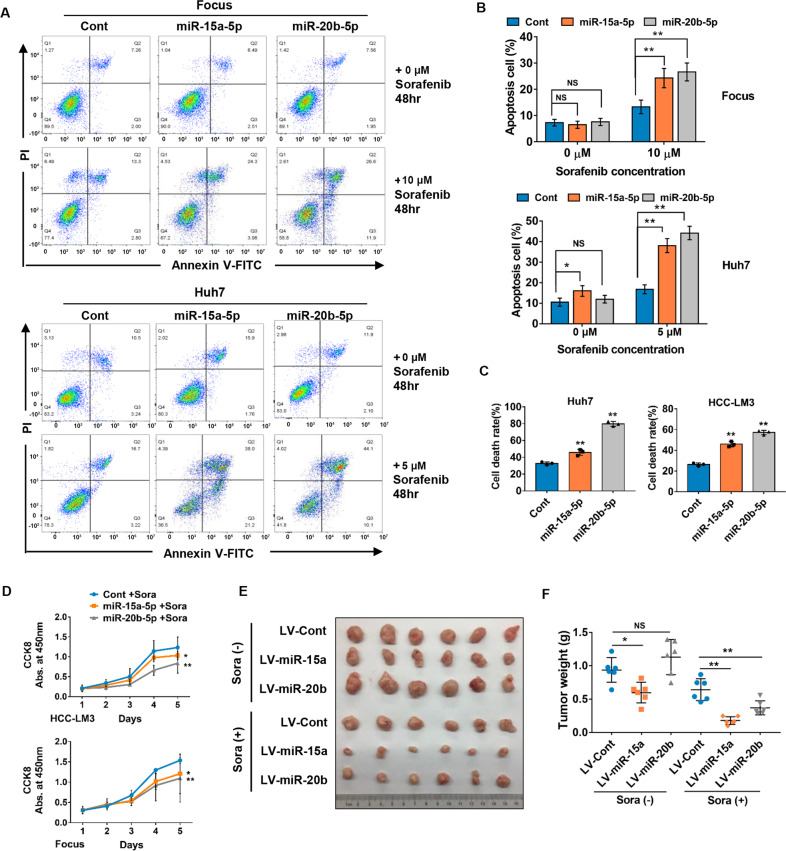
MiR-15a and miR-20b sensitize HCC cells to sorafenib. **A**, **B** Focus and Huh7 cells transfected with miR-15a or miR-20b mimics were treated with sorafenib for 48 h. Flow cytometric was performed to determine the apoptosis rates (one-way ANOVA). **C** Cell death was assessed by trypan blue staining upon sorafenib treatment in HCC cells transfected with miR-15a and miR-20b mimics. Huh7 and HCC-LM3 cells were treated with 5 μM and 10 μM sorafenib (one-way ANOVA). **D** CCK-8 assays were used to detect cell growth in HCC-LM3 and Focus cells under sorafenib (7.5 μM) treatment conditions. **E**, **F** Representative images of xenograft tumors formed in nude mice, and weights of tumors were measured after dissection (*n* = 6, one-way ANOVA). All data are shown as the mean ± SD, *p < 0.05, **p < 0.01.

To further determine the role of miR-15a and miR-20b in sorafenib-treated HCC cells, we transiently transfected their mimics into Focus and Huh7 cells, followed by addition of sorafenib. After 48 h, flow cytometric analysis was performed to detect cell apoptosis. As shown in Fig. 2A, B, overexpression of miR-15a and miR-20b in the two cells had no significant effects on cell apoptosis under normal conditions, but dramatically induced cells apoptosis under the sorafenib treatment. Similarly, a significantly elevated cell death rate was observed in HCC cells with overexpression of miR-15a or miR-20b after sorafenib treatment by trypan blue assays (Fig. 2C). Furthermore, CCK-8 cell proliferation assays showed that HCC-LM3, Focus, and Huh7 cells with upregulated expression of miR-15a or mir-20b exhibited slower cell growth upon low concentration of sorafenib treatment (Fig. 2D and Supplementary Fig. S2a). Consistently, colony formation assays demonstrated that lentivirus-mediated miR-15a or miR-20b upregulation decreased colonies number and size after sorafenib treatment, whereas had no evident effects under normal culture conditions without sorafenib treatment (Supplementary Fig. S2b-c). These findings collectively demonstrated that miR-15 or miR-20b strengthen the sensitivity of HCC cells to sorafenib.

To further verify the roles of miR-15a and miR-20b in vivo, a xenograft model of nude mice was employed. HCC-LM3 cells stably overexpressing miR-15a or miR-20b and their control cells were subcutaneously inoculated in the flanks of nude mice, respectively. When formed tumor size reached approximately 3 × 4 mm, the nude mice were randomly divided into two groups and treated with sorafenib or vehicle using gavage once every two days. Notably, we found that the tumorigenicity was mildly enhanced in HCC cells with forced expression of miR-20b in the non-sorafenib treatment group, whereas miR-15a overexpression led to opposite results, implicating the two miRNAs play distinct roles in HCC. More importantly, after sorafenib treatment, tumor size and weight of miR-15a or mir-20b overexpressed group were significantly smaller and lower in comparison with the respective control groups (Fig. 2E, F), suggesting that miR-15a and 20b function to suppress sorafenib activity of HCC.

### CDC37L1 was identified as a common target of miR-15a and miR-20b

**Fig. 3:**
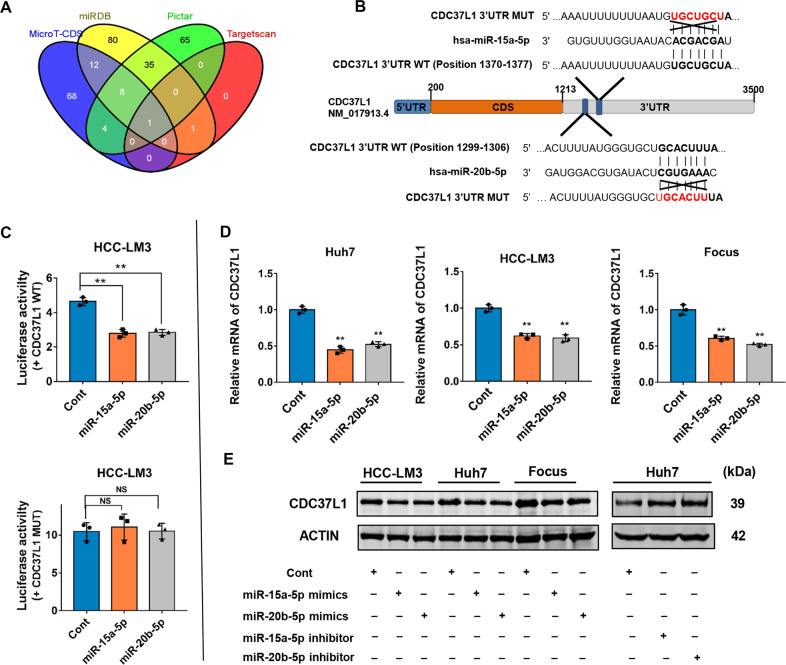
CDC37L1 is a common target of miR-15a and miR-20b. **A** Four computational algorithms predicted the potential targets of miR-15a or miR-20b. **B** Original and mutated sequences of miR-15a or miR-20b-binding elements on the 3′UTR of CDC37L1 mRNA are shown. **C** Relative luciferase activity in miR-15a or miR-20b overexpressed HCC-LM3 cells with the CDC37L1 3′UTR WT or MUT, respectively (one-way ANOVA). **D** qRT-PCR was employed to measure CDC37L1 mRNA levels in HCC cells transfected with miR-15a or 20b mimics (one-way ANOVA). **E** CDC37L1 protein levels were examined in HCC cells after transfection of miR-15a or 20b mimics or inhibitors by western blot analysis. The data are presented as the mean ± SD from three independent experiments, *p < 0.05, **p < 0.01.

MiR-15a and miR-20b belong to the microRNA family, an endogenous non-coding small RNA, which participates in various cellular life activities through negative regulation of target genes expression. Herein, to clarify the mechanisms of their effects on sorafenib resistance, we searched the potential functional targets of miR-15a and miR-20b using several miRNA target prediction algorithms including Micro T-CDS, miRDB, PicTar, and Targetscan [24–27]. Coincidentally, CDC37L1 was found to be the only shared gene among the putative targets of miR-15a and miR-20b, and it is also an important cochaperone (Fig. 3A). As described in Fig. 3B, Targetscan analysis predicted that CDC37L1 contains seed sequences of miR-15a and miR-20b in its 3′ untranslated region (3′UTR). In order to validate whether miR-15a and 20b directly target CDC37L1 mRNA, we then generated dual-luciferase reporter vectors containing a fragment of the wild-type 3′UTR of CDC37L1 mRNA (CDC37L1 WT) or a mutant 3′UTR in the seed region (CDC37L1 MUT) (Fig. 3B), and luciferase reporter assays were performed subsequently. As expected, enforced expression of miR-15a or 20b markedly limited the luciferase activity of CDC37L1-WT plasmid, whereas no significant changes were found in the luciferase activity of CDC37L1-MUT plasmid, suggesting CDC37L1 is a common direct target of miR-15a and 20b (Fig. 3C). qRT-PCR results showed that ectopic expression of miR-15a or miR-20b could effectively downregulate the mRNA level of CDC37L1 in HCC cells (Fig. 3D), and CDC37L1 protein expression was also suppressed by miR-15a or miR-20b as evidenced by western blots (Fig. 3E). Collectively, these data identified a negative regulation between miR-15a or 20b and their common targeted gene CDC37L1.

### CDC37L1 reinforced sorafenib resistance in HCC cells

**Fig. 4:**
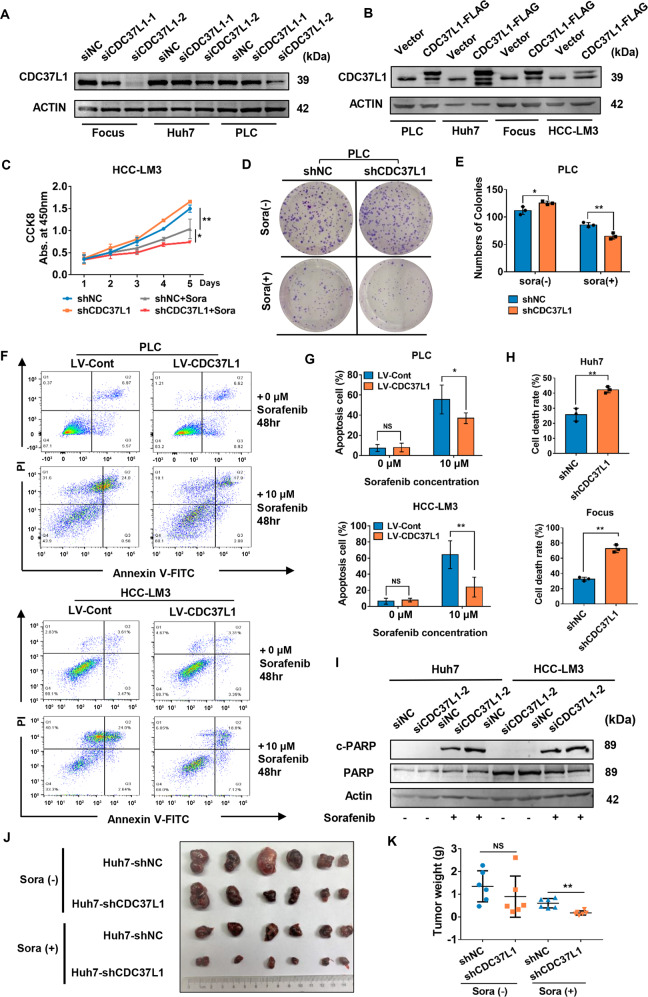
CDC37L1 reduced the anti-tumor effects of sorafenib in HCC in vitro and in vivo. **A** The knockdown efficiency of CDC37L1 in three HCC cell lines via western blotting. **B** The overexpression of CDC37L1 was detected by western blot analysis in HCC cells as indicated. **C** CCK8 assay was used to detect cell viability in HCC-LM3 cells after knockdown of CDC37L1 under normal or sorafenib (7.5 μM) treatment condition (one-way ANOVA). **D**, **E** Colony formation capability of PLC/PRF/5 cells with downregulated CDC37L1 with or without sorafenib (7.5 μM) treatment (unpaired *t* test, two-tailed). **F**, **G** Cell apoptosis rate was evaluated by flow cytometric in PLC/PRF/5 and HCC-LM3 cells with CDC37L1 overexpression under normal or sorafenib treatment condition (unpaired *t* test, two-tailed). **H** Trypan blue staining was performed to study cell death rate in the presence of sorafenib when CDC37L1 was knocked down (unpaired *t* test, two-tailed). Huh7 and Focus cells were treated with 5 and 10 μM sorafenib. **I** Expression of apoptosis-related proteins (PARP and c-PARP) were assessed in CDC37L1 knockdown cells with or without sorafenib treatment. **J**, **K** Representative images of xenograft tumors formed in nude mice, and weight of tumors was measured after dissection (unpaired *t* test, two-tailed). *p < 0.05, **p < 0.01.

Although previous studies have indicated that the mRNA level of CDC37L1 is slightly decreased in HBV-related HCC tissues, its correlation with sorafenib resistance remains unclear [28]. To examine whether CDC37L1 is involved in this process, we designed two siRNA sites targeting CDC37L1 for its knockdown and generated a plasmid for its overexpression. Western blot results proved that CDC37L1 could be successfully silenced or overexpressed in multiple HCC cell lines (Fig. 4A, B). Cell viability assays were performed with or without sorafenib treatment. As shown in Fig. 4C and Supplementary Fig. S3, cell growth rates of CDC37L1-silenced HCC-LM3, Huh7 and Focus cells were slightly higher than that of control cells, implicating an inhibitory role of CDC37L1 in HCC proliferation. As the same time, we observed that CDC37L1 silencing attenuated the proliferation ability of HCC cells in the presence of sorafenib (Fig. 4C and Supplementary Fig. S3). The similar phenotypes were also observed in colony formation assays (Fig. 4D, E). On the other hand, we evaluated the effect of CDC37L1 on sorafenib-induced cell apoptosis and death by flow cytometry analysis and trypan blue staining. Consistent with the above results, CDC37L1 significantly decreased the percentage of apoptotic cells induced by sorafenib, but exhibited no effect in the absence of sorafenib (Fig. 4F–H). Meanwhile, western blot results confirmed that the downregulation of CDC37L1 could enhance the sorafenib-induced cell apoptosis by cleaved PARP (c-PARP) expression, which reflects the degree of apoptosis (Fig. 4I). In addition, we resorted to reconfirm the above findings in vivo via the xenograft model of nude mice. As expected, the results indicated that tumor size and weight derived from CDC37L1-silenced cells were remarkably smaller and lower in response to sorafenib, while no significant changes were observed between two groups without drug treatment (Fig. 4J, K). Together, these data of in vitro and in vivo experiments implied that CDC37L1 plays a pivotal role in promoting sorafenib resistance in HCC cells.

### CDC37L1 increased the association of HSP90 and PPIA to enhance HCC cell resistance to sorafenib

**Fig. 5:**
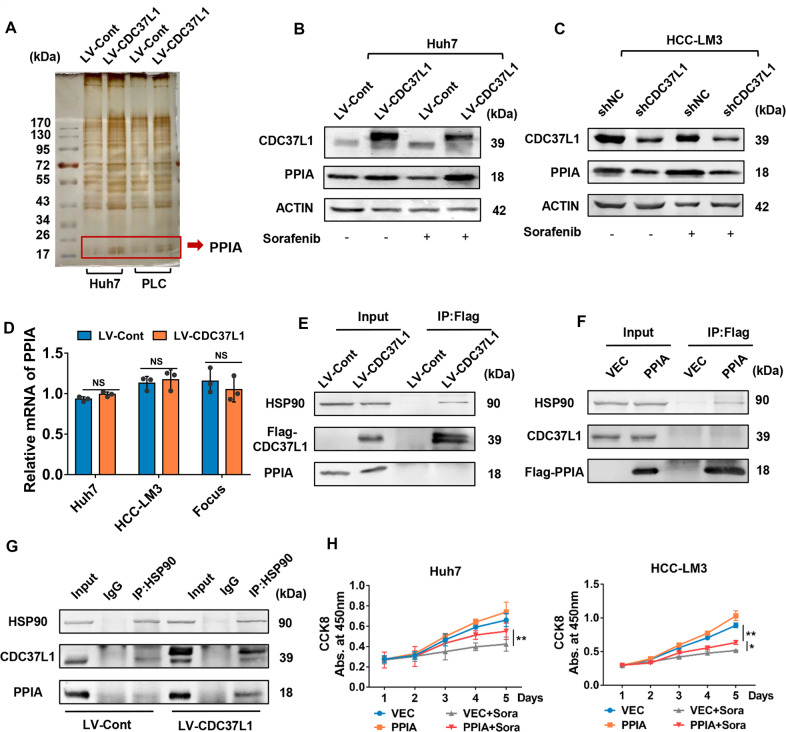
CDC37L1 enhanced HSP90/CDC37L1/PPIA complex formation. **A** The whole-cell lysates from Huh7 and PLC/PRF/5 cells with CDC37L1 stable overexpression and control cells were subjected to SDS-PAGE and visualized by sliver staining. The protein band was cut out to be identified by mass spectrometry. **B** PPIA protein expression was assessed by western blot in Huh7 cells with CDC37L1 overexpression. **C** Western blot results indicated PPIA expression in HCC-LM3 cells with CDC37L1 knockdown. **D** The mRNA level of PPIA was evaluated by qRT-PCR in CDC37L1 overexpressed cells (paired *t* test, two-tailed). **E** Co-IP assay was performed in Huh7 cells, in which exogenous FLAG-tagged CDC37L1 was introduced. HSP90, but not PPIA was co-immunoprecipitated with the anti-FLAG antibody. **F** Huh7 cells were transfected with FLAG-tagged PPIA plasmid. Cell lysates were immunoprecipitated with anti-FLAG antibody, and then the precipitates were assessed by western blotting with anti-FLAG, anti-HSP90 and anti-CDC37L1 antibodies. **G** Endogenous HSP90 was pulled down with anti-HSP90 antibody in CDC37L1 overexpressing Huh7 cells and control cells via Co-IP analysis, then CDC37L1 and PPIA were examined by western blotting. Normal IgG worked as control antibody. **H** CCK8 assay was used to analyze cell viabilities in Huh7 and HCC-LM3 cells with or without PPIA overexpression in the absence or presence of sorafenib (means ± SD, one-way ANOVA). Huh7 and HCC-LM3 cells were treated with 3 μM and 7.5 μM sorafenib. *p < 0.05, **p < 0.01.

To gain insight into the mechanism of CDC37L1 in sorafenib resistance, we carried out SDS-PAGE and silver staining assay followed by mass spectrometry to analyze the immunoprecipitated proteins of CDC37L1 in Huh7 and PLC/PRF/5 cells. A protein with a molecular weight of approximately 17 kDa came to our attention (Fig. 5A). Subsequent analysis of the band by mass spectrometry identified that PPIA might be a candidate protein affected by CDC37L1. The unique peptides of PPIA identified by mass spectrometry were shown in Supplementary Fig. S4. To confirm this finding, western blot assay was performed in HCC cells with upregulated or downregulated CDC37L1, respectively. The resulting data demonstrated that PPIA expression was regulated by CDC37L1 overexpression or knockdown with or without sorafenib treatment (Fig. 5B, C).

Previous studies have shown that CDC37L1 acts as a cochaperone to assist the heat shock protein 90 (HSP90) in regulating their client protein expression [29]. To investigate whether PPIA is involved in this mechanism, we first excluded that the regulation of PPIA by CDC37L1 at mRNA level via qRT-PCR (Fig. 5D). Next, co-immunoprecipitation (co-IP) assay was performed to detect the interaction of FLAG-tagged CDC37L1 with HSP90 and PPIA in HCC cells. Interestingly, exogenous CDC37L1 has a significant interaction with HSP90, but no interaction with PPIA (Fig. 5E). As well, exogenous PPIA was weakly immunoprecipitated with HSP90, but not CDC37L1 (Fig. 5F). Further, to strengthen the evidence for interactions among HSP90, CDC37L1, and PPIA, endogenous PPIA was immunoprecipitated in Huh7 cells with or without CDC37L1 overexpression. Western blot results indicated that both endogenous and exogenous CDC37L1 were co-immunoprecipitated with HSP90, and much more signal of PPIA was observed in CDC37L1 overexpressed cells compared with control cells, suggesting CDC37L1 may promote the interaction between HSP90 and PPIA (Fig. 5G). Functionally, we found that PPIA significantly facilitated cell viability after sorafenib treatment (Fig. 5H). These results strongly supported the notion that PPIA may mediate the effect of CDC37L1 on sorafenib resistance of HCC cells.

### CDC37L1 and PPIA were associated with poor outcomes in patients with HCC after therapy with sorafenib

**Fig. 6:**
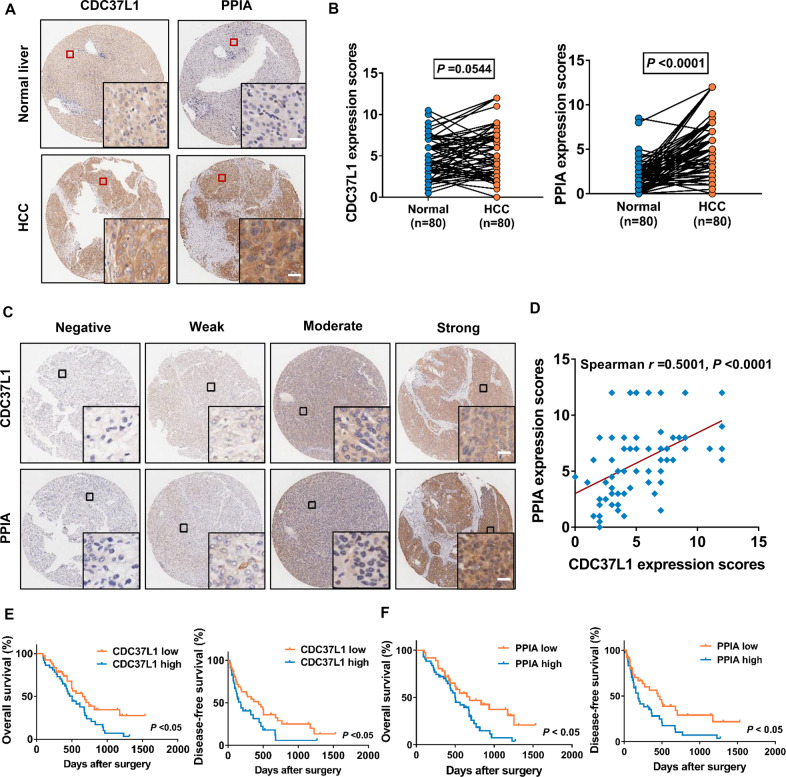
CDC37L1 and PPIA predict poor prognosis of HCC patients subjected to sorafenib therapy. **A** Representative images of IHC staining of CDC37L1 and PPIA in normal and HCC tumor tissues. **B** Quantification of CDC37L1 and PPIA protein levels in 80 pairs of normal and HCC tissues (*n* = 80, paired *t* test, two-tailed). **C** Representative images of IHC staining of CDC37L1 and PPIA in consecutive HCC tissues. **D** The expression correlation of CDC37L1 and PPIA in (**C**) was analyzed. **E**, **F** Overall survival and disease-free survival of CDC37L1 or PPIA-high and −low group patients (*n* = 80, log-rank test). Scale bars: 25 μm.

To further explore the clinical correlation between CDC37L1 and PPIA and their association with clinicopathological parameters of HCC patients, we accessed the expression of CDC37L1 and PPIA in a tissue microarray containing 80 pairs of HCC and adjacent normal tissues derived from patients who received sorafenib treatment after surgery via immunohistochemistry (IHC) staining. Statistical analysis indicated that there was no significant difference in the level of CDC37L1 between HCC tissues and non-tumor tissues, while higher expression of PPIA was observed in HCC tissues (Fig. 6A, B). Furthermore, the correlation between CDC37L1 and PPIA expression in HCC tissues was evaluated by Spearman correlation analysis, and a positive correlation between CDC37L1 and PPIA expression in HCC was obtained (Fig. 6C, D). However, no statistical difference was found between the expression of CDC37L1 or PPIA and the patients’ age, gender, grading, and tumor size and recurrence (Supplementary Table S1). In addition, we noticed that overexpression of CDC37L1 and PPIA were both significantly associated with poorer overall survival and disease-free survival of HCC patients who received sorafenib targeted therapy (Fig. 6E, F). Therefore, these clinical data revealed that CDC37L1 expression has a positive correlation with PPIA, and the two proteins may exert sorafenib resistance function in HCC.

## Discussion

MiRNAs play key roles in almost all cellular life activities, such as proliferation, differentiation, migration, metastasis, epithelial-mesenchymal transition, and apoptosis. In the current study, we identified miR-15a and miR-20b as potential biomarker for sorafenib sensitivity in HCC. Deletion or knockdown of the two miRNAs led to sorafenib resistance and overexpression of the two miRNAs enhanced sorafenib sensitivity of HCC cells. MiR-15a is regarded as a tumor suppressor by inhibiting tumor cell proliferation, migration, and invasion, and accelerating apoptosis or necroptosis [30–32]. Moreover, the association between miR-15a and immune response was found in recent years [33, 34]. Previous study also implicated that miR-15a decreased myeloma cell growth and survival by inhibiting the G1*/*S transition. Bone marrow stromal cells (BMSC) secreted IL-6 contributes to drug resistance in myeloma cells through reducing miR-15a expression [35]. Recent reports demonstrate that miR-15a plays suppressive roles in HCC development and functions as a negative regulator for pirarubicin tolerance [36]. MiR-20b was recently studied in cancers, and was recognized as either an oncogene or a tumor suppressor gene depending on the cellular environment [37, 38]. MiR-20b can suppress glioblastoma progression by regulating tumor-associated signaling pathways, including Wnt signaling and MAPK/ERK signaling [39]. One previous study implicated that miR-17 and miR-20b attenuated breast cancer resistance to Taxol by targeting nuclear receptor coactivator 3 (NCOA3) [40]. MiR-20b was also shown to have an oncogenic effect in HCC, whose expression is positively associated with TNM stage, microvascular infiltration, and tumor recurrence [41]. However interestingly, in the context of sorafenib resistance, our data reveal that miR-15a and miR-20b have the same roles in sorafenib response.

In general, miRNAs exert their roles by post-transcriptional regulation in mRNA degradation or translation. Considering miR-15a and miR-20b have the similar phenotypes upon sorafenib treatment, we performed a bioinformatics analysis and found a common target of the two miRNAs, CDC37L1, which belongs to cell division cycle 37 (CDC37) family. Like CDC37, CDC37L1 is a cytoplasmic protein that exists in complex with HSP90 as well as several other proteins involved in HSP90-mediated protein folding [29, 42]. There is growing concern on studying HSP90-cochaperone-client complex and targeting HSP90 to overcome anti-cancer drugs resistance has been a promising strategy [43, 44]. 17-AAG, an HSP90 inhibitor, has been implicated synergistic effects in killing cancer cells when combined with Taxol in breast cancer via downregulating Akt and Her2 expressions [45]. Importantly, a study demonstrates that HSP90 inhibitor AUY922 could synergistically strengthen the toxicity of sorafenib in HCC cells [46]. Unexpectedly, our western blot results indicate that CDC37L1 has no obvious effect on HSP90 protein expression (data not shown).

Through molecular biological approaches, we demonstrate that CDC37L1 plays a role of cochaperone in enhancing the interaction between HSP90 and PPIA, which benefits to the stability of PPIA. PPIA is a central enzyme in the protein-folding response pathway, and it has been identified as a potential target for improving drug resistance in different tumors including HCC [47, 48]. Subsequent functional assays proved that PPIA overexpression confers HCC cells to sorafenib resistance. This is consistent with the finding of a recent study that PPIA inhibits the sensitivity of multiple myeloma tumor cells to proteasome inhibitors, which was identified through single-cell sequencing in relapsed multiple myeloma patients [49]. In addition, our IHC results also support the conclusion that CDC37L1 and PPIA contribute to sorafenib resistance. Therefore, PPIA may be a key client protein for the function of HSP90/CDC37L1 to enhance sorafenib resistance.

In the present study, our data demonstrate that downregulation of miR-15a and miR-20b facilitate sorafenib resistance to HCC through reinforcing the binding correlation of HSP90 with PPIA under the assistance of CDC37L1, which contribute to understand the mechanism of drug resistance and overcome tumor resistance.

## Materials and methods

### Cell culture and culture conditions

Human HCC cell lines Huh7, Focus, and PLC/PRF/5 were purchased from Cell Bank of Chinese Academy of Science, Shanghai, China. HCC-LM3 cells were kindly provided by Liver Cancer Institute of Zhongshan Hospital, Shanghai Fudan University, China. Cells were cultured in Dulbecco’s modified Eagle’s medium (DMEM) (Solarbio, Beijing, China) supplemented with 10% fetal bovine serum (FBS) (Corning, USA) and 1% Penicillin/Streptomycin (M&C Gene Technology Ltd., Beijing, China) at 37 °C under a 5% CO_2_ atmosphere.

### Genome-wide Cas9-mediated knockout screen

The Human CRISPR Knockout Pooled Library (GeCKO v2, purchased from Genechem, Shanghai, China) was used to screen the critical genes involving in sorafenib resistance. In detail, HCC-LM3 cells were infected with GeCKO v2 lentiviral sgRNA libraries followed by puromycin selection for 3 weeks. Next, these cells were divided into control or sorafenib group, and cells in sorafenib group were treated with sorafenib (7.5 μM) for 2 weeks, and the remaining cells were considered as sorafenib-resistant. The cells with or without sorafenib treatment were harvested to extract genomic DNA by TIANamp Genomic DNA Kit (TIANGEN Bio, China) and amplify sgRNA region using PCR technology, after which the enrichment of sgRNAs was analyzed by Next-Generation Sequencing (NGS) analysis (OE Biotech, Shanghai, China). Before deep sequencing, the CRISPR/Cas9 screen was performed three times to generate biological replicates.

### Plasmids, siRNAs, and cell transfection

The cDNA of CDC37L1 (GenBank Accession Number: NM_017913.4) was cloned into pEX-3 vector to generate CDC37L1 expression construct, which was purchased from GenePharma (Shanghai, China). pEnter-PPIA plasmid (GeneBank Accession Number: NM_021130) was purchased from Vigene Biosciences (Shandong, China). Two plasmids were both fused with a 3×FLAG tag at C-terminal. Small interfering RNA (siRNA, GenePharma, Shanghai, China) specifically against BLNK, ADCK4, SERHL2, THPO, MEX3B, MAGEA11, MRAS, CDCA7L, and CDC37L1 was used for loss-of-function experiments. The sense sequences are listed in Supplementary Table S2. According to deep sequence analysis of the online database miRBase (www.mirbase.org), 99.86% miR-15a-5p reads and 0.13% miR-15a-3p reads in miR-15a were found. And 96.48% miR-20b-5p reads and 3.51% miR-20b-3p reads in miR-20b were detected. These data suggest that miR-15a-5p and miR-20b-5p are dominant forms generated from their precursors (pre-miR-15a and pre-miR-20b). Therefore, we chose miR-15a-5p and miR-20b-5p as the object of subsequent researches (mimics and inhibitors). miR-15a-5p or miR-20b-5p mimic/negative control mimic and miR-15a-5p or miR-20b-5p inhibitor/negative control inhibitor were purchased from GenePharma, Shanghai, China. Cell transfections in this study were performed by the Lipofectamine 3000 Kit (Invitrogen, USA) according to the manufacturer’s protocols.

### Construction of miR-15a and miR-20b knockout cell line by CRISPR/Cas9-mediated gene editing

HCC-LM3 cells were plated in 35 mm dishes to about 80% confluence, and transfected with sg-miR-15a plasmid, sg-miR-20b plasmid, and control vector, respectively. The plasmids used to knock out the two miRNAs were obtained from YSY Biotech, Nanjing, China. The targeting sequences for miR-15a: 5′-TGTGCTGCTACTTTACTCCA-3′; for miR-20b: 5′-TACCAAAGTGCTCATAGTGC-3′. Cells were then selected by puromycin ( /ml) for three days. The remaining cells were seeded into 96-well plates by serial dilution for single colonies. After three weeks, the established knockout cell lines were identified by qRT-PCR and DNA sequencing.

### Lentiviral packaging and transduction

For overexpression of miR-15a or miR-20b, lentiviral particles containing miR-15a or miR-20b precursor sequence were packaged and purchased from Shanghai Tuzhu biotech, China. CDC37L1 overexpression lentivirus (LV-CDC37L1) and knockdown lentivirus (LV-shCDC37L1, based on siCDC37L1-2 sequence) were obtained from GenePharma, Shanghai, China. Lentivirus transduction was carried out at MOI (multiplicity of infection) of 10–20 for each cell line. Stably transduced cells were generated by puromycin selection.

### Quantitative real-time PCR (qRT-PCR)

Total RNA was extracted from HCC cells using TRIzol reagent (Invitrogen, USA) and reversely transcribed into cDNA using a PrimeScript^TM^ RT Reagent Kit (TaKaRa, Japan). The mRNA level of CDC37L1 and PPIA, the expression level of miR-15a and miR-20b were assessed by qRT-PCR on an ABI Q6 Real-time PCR system (ABI, CA, USA) according the manufacturer’s instructions. Each sample was normalized to β-actin or U6 snRNA prior to comparative analysis by 2^−△△Ct^ method. All experiments were performed in triplicate and repeated at least three times. The primers used in this study were listed in Supplementary Table S3.

### Cell proliferation assay

A Cell Counting Kit-8 assay (CCK-8) assay was used to assess HCC cell proliferation. Briefly, HCC cells were seeded in a 96-well plate at 4000 cells per well, then cells were treated with sorafenib (#8705, Cell Signaling Technology, MA, USA) in proper concentration after 24 h. The concentration of the sorafenib used to treat different HCC cell lines was determined based on estimated IC50 values of respective cell lines (Supplementary Fig. S5). Due to different drug susceptibility of cells, IC50 values differed across various cell lines that we used. The specific concentration is described in the respective figure legend. Importantly, the moment that sorafenib was added in wells was considered as the first day of the experiment. Then 10 µl CCK-8 reagent (Dojindo Laboratories, Japan) was added to each well, cells were inoculated 75 min at 37 °C and cellular absorbance at 450 nm was measured using a SpectraMax M5 Microplate Reader (Molecular Devices, CA, USA). All experiments were performed in triplicate and repeated at least 3 times independently.

### Colony formation assay

Briefly, HCC cells were plated in 6-well plates at a density of 1000 per well. Cells were then treated or untreated with sorafenib when cells were attached to a culture dish. After incubation for around 2–3 weeks, the cells were fixed with 4% paraformaldehyde and subsequently stained with 0.5% crystal violet. The mean numbers of colonies were calculated. And independent assays were performed in three times.

### Flow cytometric analysis of apoptosis

Apoptotic cells were detected using an Annexin V-FITC kit (Dojindo Laboratories, Japan) 48 h after treatment with or without sorafenib according to the manual instruction. Cell apoptosis rate was assessed by flow cytometry in triplicate (Arial III, BD Biosciences, CA, USA). The experiments were repeated at least three times.

### Trypan blue staining assay

Cell survival analysis was performed in sorafenib-treated HCC cells using 0.4% Trypan blue dye in PBS buffer. The cells that did not take up the dye were considered viable, while those which took the dye were considered non-viable, and cell death rate was ultimately calculated from three independent experiments.

### Western blot analysis

Total protein was extracted from HCC cell lines using RIPA lysis buffer (Merck Millipore, MA, USA) and quantified by BCA assay (Takara Bio, Kyoto, Japan). The samples were subjected to SDS-PAGE and immediately transferred to nitrocellulose filter (NC) membranes (0.45 μm, Bio-Rad, CA, USA), then incubated with primary antibodies. Expression of proteins was detected by the Odyssey Infrared Imaging System (Li-COR, NE, USA). The primary antibodies used in this work are as follows: Anti-CDC37L1 (1:500, 16293-1-AP, Proteintech, Wuhan, China), anti-β-actin (1:1000, sc-81178, Santa Cruz Biotechnology, TX, USA), anti-PARP (1:1000, 9532S, Cell Signaling Technology, MA, USA), anti-cleaved PARP (1:500, 5625 S, Cell Signaling Technology, MA, USA), anti-HSP90 (1:500, 13171-1-AP, Proteintech, Wuhan, China), anti-PPIA (1:1000, ab41684, Abcam, MA, USA).

### Co-immunoprecipitation (Co-IP) analysis

HCC cells were seeded in 10-cm dishes for the requirement of this assay. Then cells were lysed with lysis buffer (50 mM TrisHCl, pH 7.5, 100 mM NaCl, 0.5% Nonidet P-40) supplemented with a protease inhibitor cocktail. Cell lysates were incubated with anti-Flag, anti-HSP90 (66008-3-Ig or 13171-1-AP, Proteintech, China) or normal rabbit immunoglobulin G (IgG) (2729, Cell Signaling Technology, MA, USA) at 4 °C for 2 h, and Dynabeads^TM^ Protein G (#10003D, Thermo Fisher Scientific, USA) were added to lysates overnight at 4 °C. The beads were washed four times with the lysis buffer described above and subjected to SDS-PAGE, followed by Western blot analysis.

### Silver staining and mass spectrometry

The silver staining assay was carried out using Fast Silver Stain kit (P0017S, Beyotime Biotech, Beijing, China) according to the manufacturer’s instructions. HCC cells transduced with LV-CDC37L1 or LV-Cont were collected, washed, lysed, and then suffered SDS-PAGE and silver stain detection. The selected bands were excised and identified via mass spectrometry in Luming Biotech, Shanghai, China.

### Luciferase activity assay

Full-length sequence of CDC37L1 3′UTR was cloned into pGL3-promoter vector (Promega, WI, USA), designated pGL3-CDC37L1-WT. miR-15a and miR-20b binding sites were then mutated by site-directed mutagenesis in this construct to generate pGL3-CDC37L1-MUT. Two luciferase reporter plasmids were confirmed by DNA sequencing. Prior to luciferase activity assay, HCC cells with stable miR-15a or 20b expression were seeded into 24-wells plates, co-transfected with the plasmid pGL3-CDC37L1-WT (or mutant plasmid) and pRL-SV40 by Lipofectamine 3000 and incubated for 24 h. The luciferase activity was quantified by Dual-Luciferase reporter assay system (Promega, WI, USA) in accordance with the manufacturer’s protocols. Each experiment was repeated three times.

### Animal model

HCC-LM3 cells were infected with miR-15a or miR-20b overexpression lentiviruses or negative control separately, and Huh7 cells were stably infected with CDC37L1-knockdown or negative control lentiviruses. Next, HCC-LM3 (2 × 10^6^) and Huh7 (3.0 × 10^6^) cells were counted and subcutaneously injected into male NOD/SCID mice (4–5 weeks old, SLAC Laboratory Animal Co. Shanghai, China). When tumor size reached approximately 3 × 4 mm, the nude mice were divided into two groups: the experimental group and the control group. The experimental groups (+sora) received intragastrically a dose of 50 mg/kg body weight of sorafenib every 2 days, whereas the mice in control groups (-sora) received only corresponding vehicles. Three to four weeks later, the mice were sacrificed and generated tumors were weighted. All animal handling and experimental procedures were received approval by the Institutional Animal Care and Use Committee (IACUC) at Shanghai East Hospital, Tongji University.

### Human tissue microarray (TMA) and immunohistochemistry

A tissue microarray (LVC1607) containing human HCC samples and the paired adjacent normal tissues was obtained from Shanghai SuperBiotek CO., LTD. Specially, the HCC patients were treated with sorafenib after surgery. Standard immunohistochemistry (IHC) staining procedures were performed with specific antibodies against CDC37L1 (16293-1-AP, Proteintech, China) and PPIA (ab41684, Abcam, USA), respectively. A semiquantitative score system was performed by two pathologists independently in a double-blinded manner to evaluate CDC37L1 or PPIA expression. The positive proportion of staining was classified as 0, <5%; 1, 5–25%; 2, 25–50%; 3, 50–75%; 4, 75–100%. The staining intensity was then classified as 0, negative; 1, weak; 2, moderate; 3, strong. The total score of CDC37L1 or PPIA staining was determined through proportion scores multiplied by intensity scores. The use for human sample was approved by the Medical Ethics Committees of Shanghai East Hospital, Tongji University School of Medicine.

### Statistical analysis

Quantitative data were shown as means ± standard deviation (SD), and all statistical analyses were performed using GraphPad Prism 7.0 software. In detail, two-tailed student’s *t*-test was used to calculate two-group comparison; one-way ANOVA was carried out to analyze multiple-group comparison; the survival analysis was applied to Kaplan–Meier method and compared by the log-rank test; Spearman correlation test was performed to examine the correlation analysis. *P* < 0.05 was considered statistically significant (**p < 0.01, *p < 0.05).

## Supplementary information

Supplementary information

Supplementary Table S1

Supplementary Table S2

Supplementary Table S3

Fig S1

Fig S2

Fig S3

Fig S4

Fig S5

Original Data File

## Data Availability

All data and material in the study are available when requested. All data analyzed and generated in this study are included in this published article and its supplementary information files.
