# Supplementary material for: miR-15a and miR-20b sensitize hepatocellular carcinoma cells to sorafenib through repressing CDC37L1 and consequent PPIA downregulation
Source: Cell Death Discov. 2022 Jun 27;8:297. doi: 10.1038/s41420-022-01094-2 (PMC9237098; doi:10.1038/s41420-022-01094-2)
Supplement: Supplementary file 1 — Supplementary information [file 41420_2022_1094_MOESM1_ESM.docx]

**miR-15a and miR-20b sensitize hepatocellular carcinoma cells to sorafenib through repressing CDC37L1 and consequent PPIA downregulation**

Li Li^1, a^, Shijun Yu^1, a^, Jingde Chen^1^, Ming Quan^1^, Yong Gao^1^, Yandong Li^1,2^

^1^Department of Oncology, Shanghai East Hospital, Tongji University School of Medicine, Shanghai 200120, China

^2^Research Center for Translational Medicine, Shanghai East Hospital, Tongji University School of Medicine, Shanghai 200120, China

**Correspondence:** Yandong Li and Yong Gao, Department of Oncology, Shanghai East Hospital, Tongji University School of Medicine, 150 Ji-Mo Rd., Shanghai 200120，China. Phone: 86-21-61569884; Fax: +86-21-58798999; E-mail: [yandongli2009@gmail.com](mailto:yandongli2009@gmail.com) and [drgaoyong@tongji.edu.cn](mailto:drgaoyong@tongji.edu.cn)

^a^Li Li and Shijun Yu contributed equally to this work.

**Supplementary Information**

**Supplementary Information contains Supplementary Tables, Supplementary Figures and Supplementary Material.**

**Supplementary Table S1** Correlation of CDC37L1 or PPIA expression with clinicopathological features of HCC patients (n=80) from HCC tissue microarray.

**Supplementary Table S2** siRNA sequences for knockdown used in this study.

**Supplementary Table S3** Primers for qRT-PCR used in this study.

**Supplementary Fig. S1 Deletion of miR-15a or miR-20b enhances the sorafenib resistance of HCC-LM3 cells.** **a** Colony formation capability of HCC-LM3 cells with miR-15a or miR-20b deletion via CRISPR/Cas9 gene editing in the presence or absence of sorafenib (7.5 μM). Representative pictures were shown. **b** The data are presented means ± SD, unpaired t test, two-tailed. *p < 0.05, **p < 0.01.

**Supplementary Fig. S2** **Overexpression of miR-15a or miR-20b attenuates the resistance of HCC cells to sorafenib.** **a** CCK-8 assay was used to detect cell growth in Huh7 cells under sorafenib (3 μM) treatment conditions (means ± SD, one-way ANOVA). **b** Representative pictures of colony formation assay. **c** Colony formation capability of HCC-LM3 cells with lentivirus-mediated overexpression of miR-15a or miR-20b in the presence or absence of sorafenib (7.5 μM). The data are presented means ± SD, unpaired t test, two-tailed. *p < 0.05, **p < 0.01.

**Supplementary Fig. S****3 Knockdown of CDC37L1 increases cell sensitivity to sorafenib.** CCK8 assay was used to detect cell viability in Huh7 and Focus cells after knockdown of CDC37L1 under normal or sorafenib treatment condition. Huh7 and Focus cells were treated with 3 μM and 7.5 μM sorafenib (means ± SD, one-way ANOVA). *p < 0.05, **p < 0.01.

**Supplementary Fig. S4** The unique peptide of PPIA were identified by mass spectrometry.

**Supplementary Fig. S5 The sensitivity of different HCC cell lines towards sorafenib.** HCC cells lines were treated with sorafenib (0 to 50 μM) for 48 hours and cell viability was measured by CCK8 assay.

**Supplementary Material** The full uncropped and unedited version of western blots.
