# Supplementary material for: miR-15a and miR-20b sensitize hepatocellular carcinoma cells to sorafenib through repressing CDC37L1 and consequent PPIA downregulation
Source: Cell Death Discov. 2022 Jun 27;8:297. doi: 10.1038/s41420-022-01094-2 (PMC9237098; doi:10.1038/s41420-022-01094-2)
Supplement: Supplementary file 2 — Supplementary Table S1 [file 41420_2022_1094_MOESM2_ESM.docx]

| **Clinicopathological variables** | ***n*** | **CDC37L1 expression** | |  | **PPIA expression** | |  |
| --- | --- | --- | --- | --- | --- | --- | --- |
|  |  | **Low** | **High** | ***P* value** | **Low** | **High** | ***P* value** |
| **Age** | | | | | | | |
| <60 | 69 | 39 | 30 | 0.213 | 31 | 38 | 0.552 |
| ≥60 | 11 | 4 | 7 |  | 6 | 5 |  |
| **Gender** | | | | | | | |
| Female | 5 | 3 | 2 | 0.772 | 2 | 3 | 0.772 |
| Male | 75 | 40 | 35 |  | 35 | 40 |  |
| **Grading** | | | | | | | |
| I,II | 5 | 1 | 4 | 0.118 | 3 | 2 | 0.524 |
| III,IV | 75 | 42 | 33 |  | 34 | 41 |  |
| **Tumor size (cm)** | | | | | | | |
| ≤5 | 30 | 17 | 13 | 0.685 | 14 | 16 | 0.954 |
| >5 | 50 | 26 | 24 |  | 23 | 27 |  |
| **Tumor recurrence** | | | | | | | |
| Yes | 51 | 29 | 22 | 0.459 | 22 | 29 | 0.459 |
| No | 19 | 14 | 15 |  | 15 | 14 |  |

**Supplementary Table S1:** **The correlation between the clinicopathologic characteristics and CDC37L1 or PPIA expression (n = 80)**

Chi-square test; ^*^*P* < 0.05 indicates a significant association among the variables.
