# Supplementary material for: miR-15a and miR-20b sensitize hepatocellular carcinoma cells to sorafenib through repressing CDC37L1 and consequent PPIA downregulation
Source: Cell Death Discov. 2022 Jun 27;8:297. doi: 10.1038/s41420-022-01094-2 (PMC9237098; doi:10.1038/s41420-022-01094-2)
Supplement: Supplementary file 3 — Supplementary Table S2 [file 41420_2022_1094_MOESM3_ESM.docx]

| Supplementary Table S2: the siRNA sequences used in this study for gene knockdown   \| Name \| Sequence \| \| --- \| --- \| \| siNC \| Sence: 5’- UUCUCCGAACGUGUCACGUTT-3’ \| \| siBLNK \| Sence: 5’- CCACUGGACAGUUAUUCGUTT-3’ \| \| siADCK4 \| Sence: 5’- CUGCUGUAGUGCCUCUUAUUU-3’ \| \| siSERHL2 \| Sence: 5’- CUCCUGGCUAAUAAUAAAUUU-3’ \| \| siTHPO \| Sence: 5’- CCUCAGUAAACUGCUUCGUTT-3’ \| \| siMEX3B \| Sence: 5’- GACGGAAUUACAACCAUAAUU-3’ \| \| siMAGEA11 \| Sence: 5’- GGGAGUUUAAGACCUACAAUU-3’ \| \| siMRAS \| Sence: 5’- GUGCCUCUCUGAUACGAAUUU-3’ \| \| siCDCA7L \| Sence: 5’-CCGCUAAAUUUGCGGAAGATT-3’ \| \| siCDC37L1-1 \| Sence: 5’- GGUGAUCAGACACUUAGUATT-3’ \| \| siCDC37L1-2 \| Sence: 5’- AGCAGAGGAAGAAGGUUAUTT-3’ \| |
| --- | --- | --- | --- | --- | --- | --- | --- | --- | --- | --- | --- | --- | --- | --- | --- | --- | --- | --- | --- | --- | --- | --- | --- | --- |
