# Supplementary material for: miR-15a and miR-20b sensitize hepatocellular carcinoma cells to sorafenib through repressing CDC37L1 and consequent PPIA downregulation
Source: Cell Death Discov. 2022 Jun 27;8:297. doi: 10.1038/s41420-022-01094-2 (PMC9237098; doi:10.1038/s41420-022-01094-2)
Supplement: Supplementary file 4 — Supplementary Table S3 [file 41420_2022_1094_MOESM4_ESM.docx]

Supplementary Table S3: The primer sequences used in this study for qRT-PCR

| Gene | Sequence |
| --- | --- |
| CDC37L1 | F: 5’- GGTGTTTTCACCTGGAAGC -3’ |
|  | R: 5’- CTCTTGGATCCACATTACAG -3’ |
| PPIA | F: 5’- CCCACCGTGTTCTTCGACATT-3’ |
|  | R: 5’- GGACCCGTATGCTTTAGGATGA -3’ |
| β-actin | F: 5’- CCTGGCACCCAGCACAATG -3’ |
|  | R: 5’- GGGCCGGACTCGTCATACT-3’ |
| Hsa-miR-15a | F: 5’- GAGTAGCAGCACATAATGG -3’ |
|  | R: 5’- GTGCAGGGTCCGAGGT -3’ |
|  | RT: 5’- GTCGTATCCAGTGCAGGGTCCGAGGTATTC  GCACTGGATACGACACAAAC -3’ |
| Hsa-miR-20b | F: 5’- GAGcaaagtgctcatagtg -3’ |
|  | R: 5’- GTGCAGGGTCCGAGGT -3’ |
|  | RT: 5’-GTCGTATCCAGTGCAGGGTCCGAGGTATTC  GCACTGGATACGACctacct -3’ |
| U6 | F: 5’- CTCGCTTCGGCAGCACA -3’ |
|  | RT: 5’- AACGCTTCACGAATTTGCGT -3’ |
|  |  |
