# Supplementary figures and images for: miR-15a and miR-20b sensitize hepatocellular carcinoma cells to sorafenib through repressing CDC37L1 and consequent PPIA downregulation
Source: Cell Death Discov. 2022 Jun 27;8:297. doi: 10.1038/s41420-022-01094-2 (PMC9237098; doi:10.1038/s41420-022-01094-2)

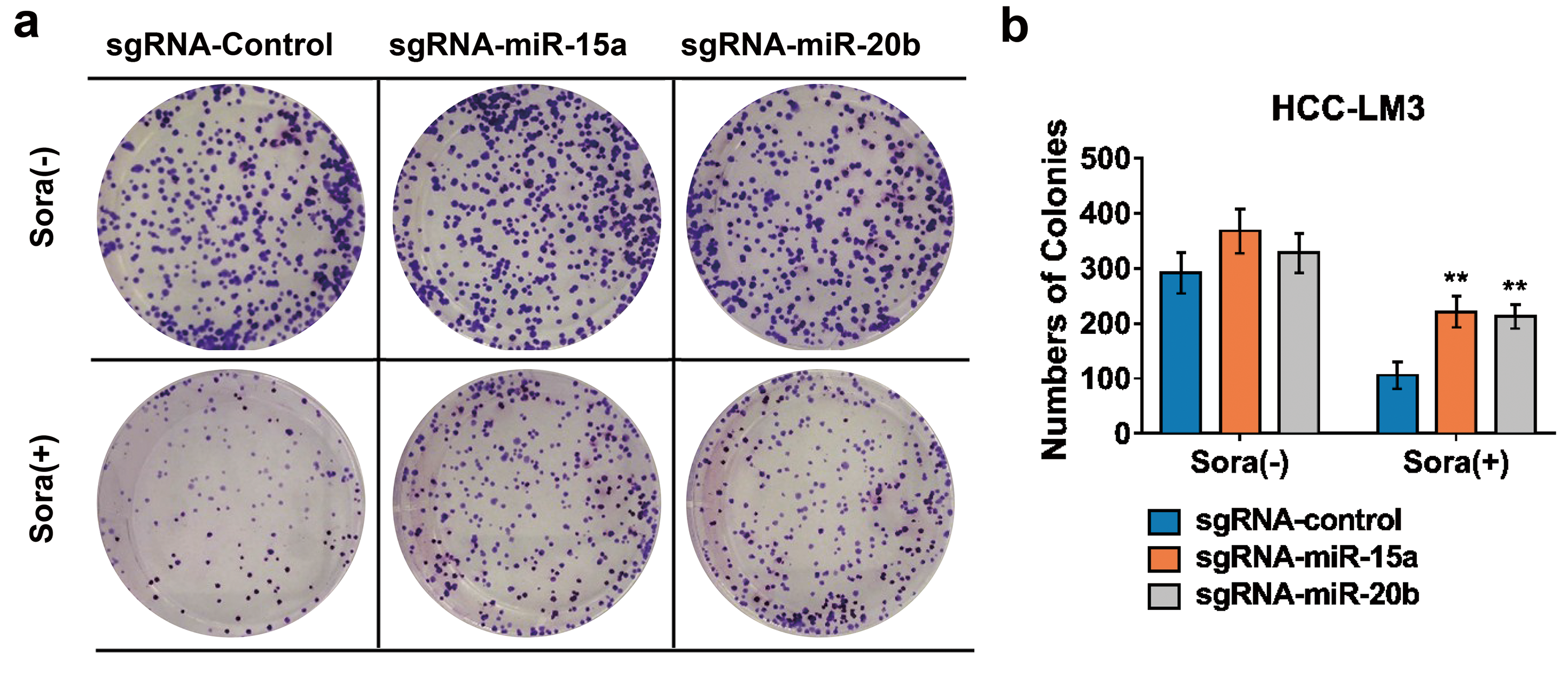

Supplement: Supplementary file 5 — Fig S1 [file 41420_2022_1094_MOESM5_ESM.tif]

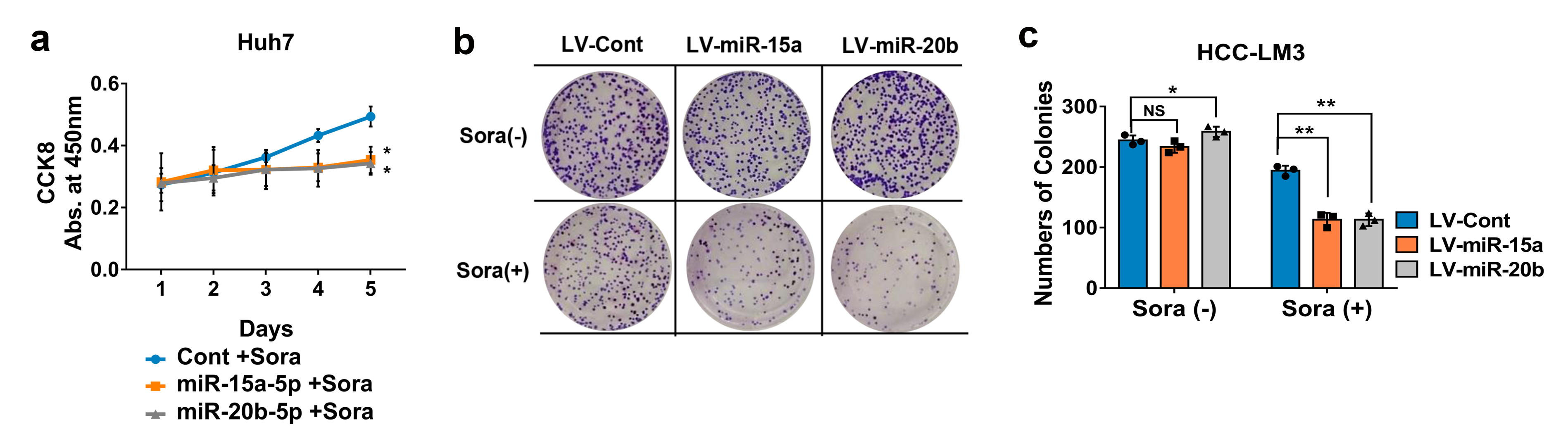

Supplement: Supplementary file 6 — Fig S2 [file 41420_2022_1094_MOESM6_ESM.tif]

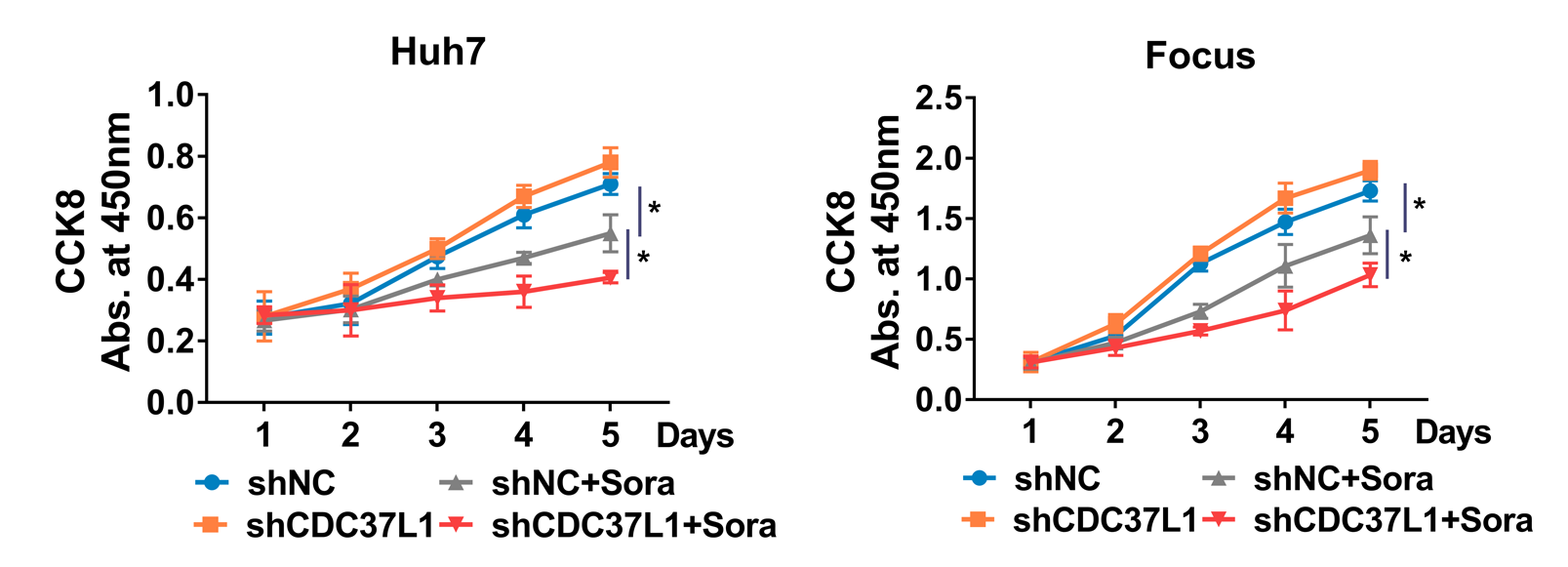

Supplement: Supplementary file 7 — Fig S3 [file 41420_2022_1094_MOESM7_ESM.tif]

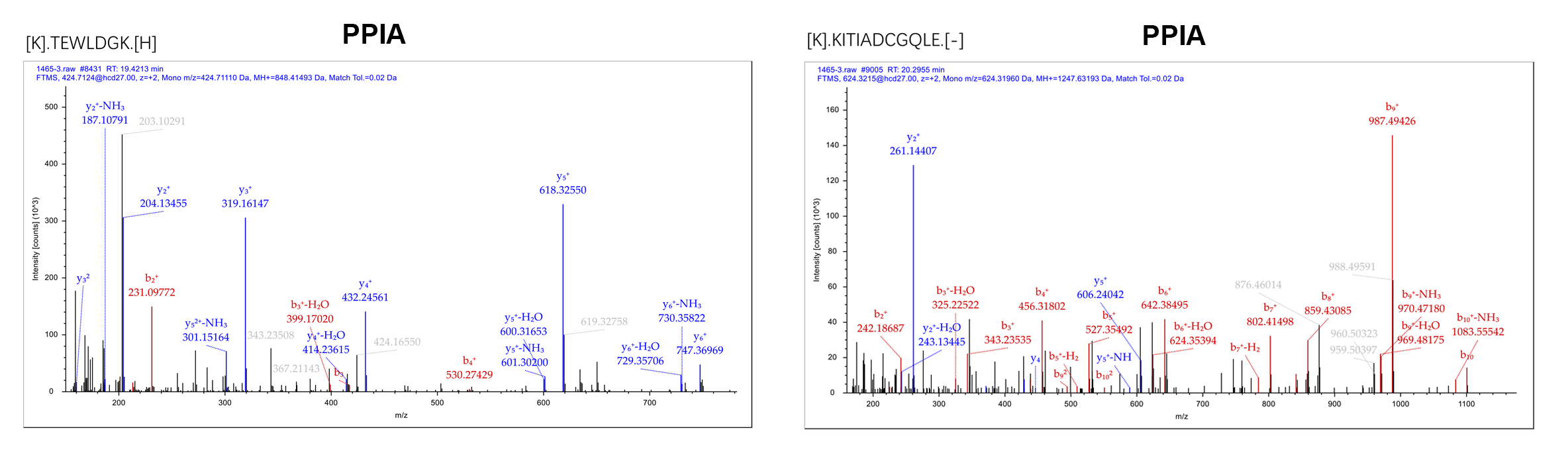

Supplement: Supplementary file 8 — Fig S4 [file 41420_2022_1094_MOESM8_ESM.tif]

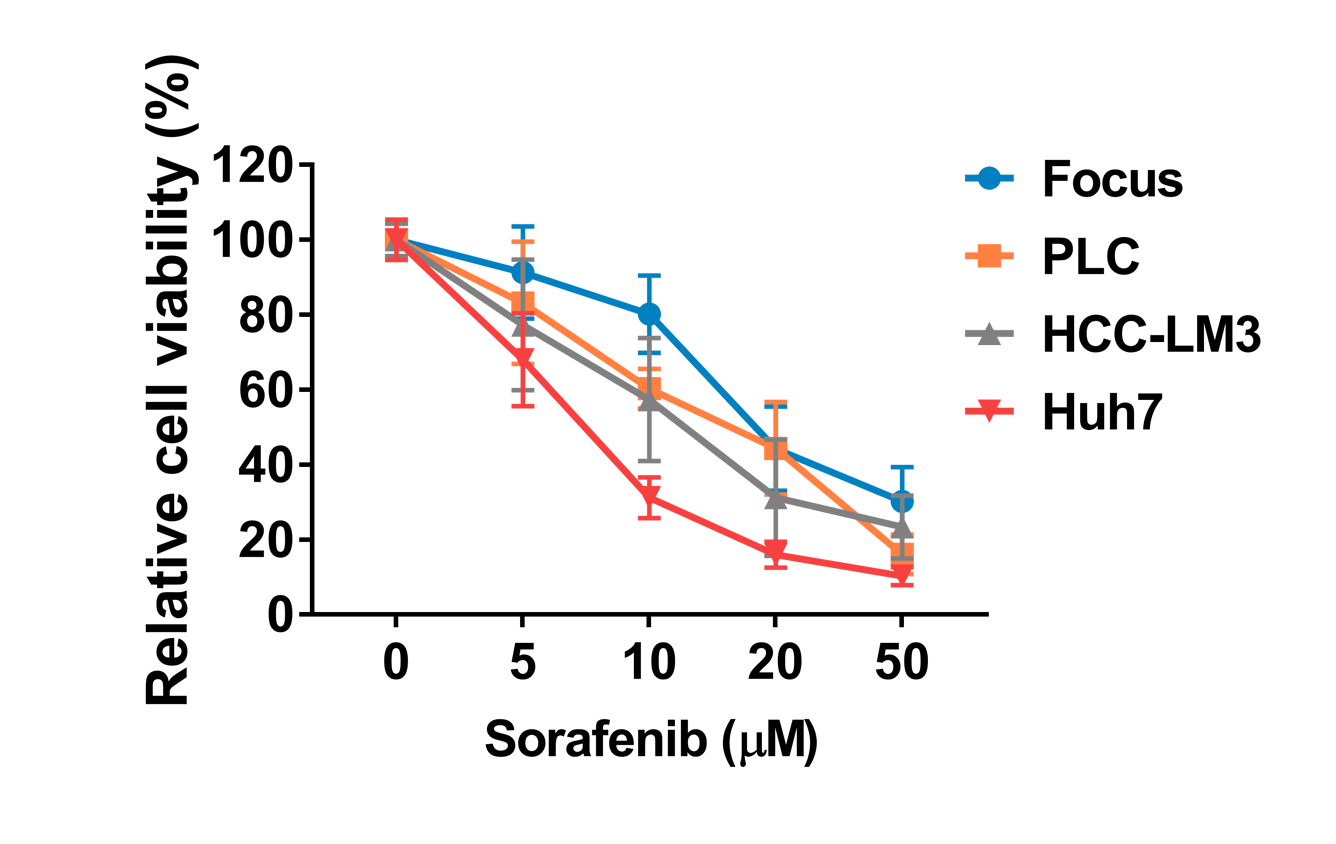

Supplement: Supplementary file 9 — Fig S5 [file 41420_2022_1094_MOESM9_ESM.tif]
