# Supplementary material for: miR-15a and miR-20b sensitize hepatocellular carcinoma cells to sorafenib through repressing CDC37L1 and consequent PPIA downregulation
Source: Cell Death Discov. 2022 Jun 27;8:297. doi: 10.1038/s41420-022-01094-2 (PMC9237098; doi:10.1038/s41420-022-01094-2)
Supplement: Supplementary file 10 — Original Data File [file 41420_2022_1094_MOESM10_ESM.pdf]

Supplementary Material

The full uncropped and unedited version of western blots.

fig.3

e

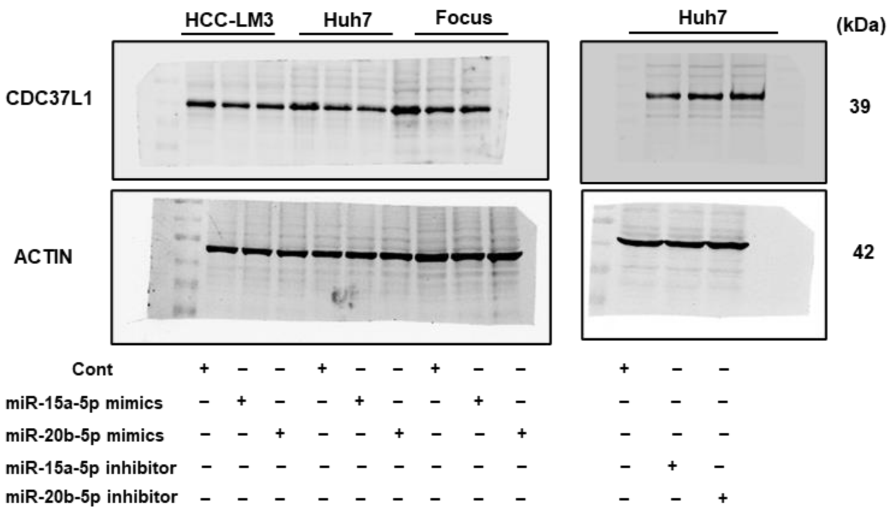

fig.4

a

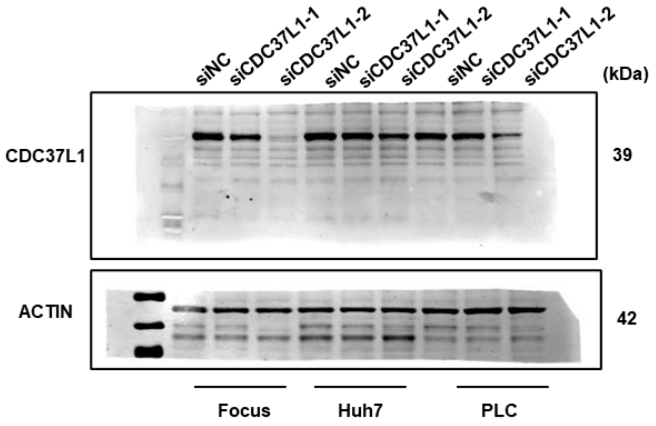
siNC
siCDC37L1-1
siCDC37L1-2
siNC
siCDC37L1-1
siCDC37L1-2
siNC
siCDC37L1-1
siCDC37L1-2
(kDa)
CDC37L1
39
ACTIN
42
Focus
Huh7
PLC

b

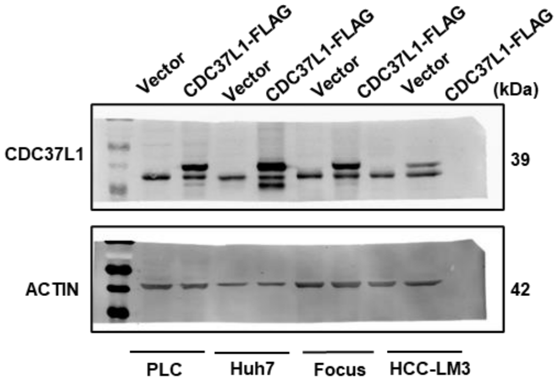
Vector
CDC37L1-FLAG
Vector
CDC37L1-FLAG
Vector
CDC37L1-FLAG
Vector
CDC37L1-FLAG
(kDa)
CDC37L1
39
ACTIN
42
PLC
Huh7
Focus
HCC-LM3

i

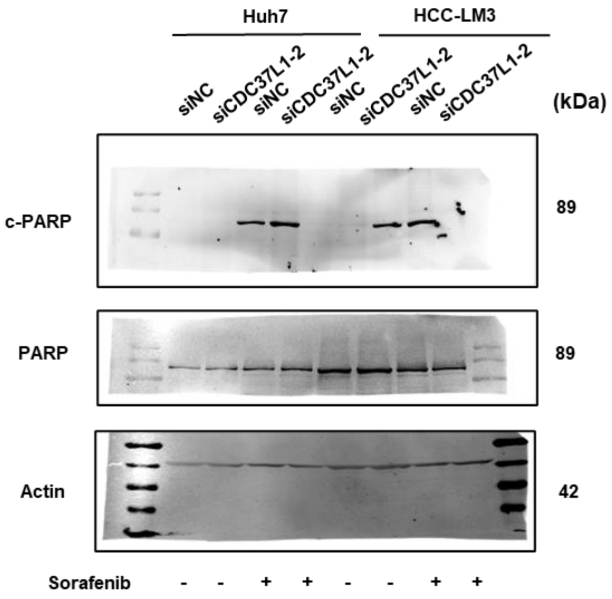
Huh7
HCC-LM3
siNC
siCDC37L1-2
siNC
siCDC37L1-2
siNC
siCDC37L1-2
siNC
siCDC37L1-2
(kDa)
c-PARP
89
PARP
89
Actin
42
Sorafenib
-
-
+
+
-
-
+
+

fig.5

b

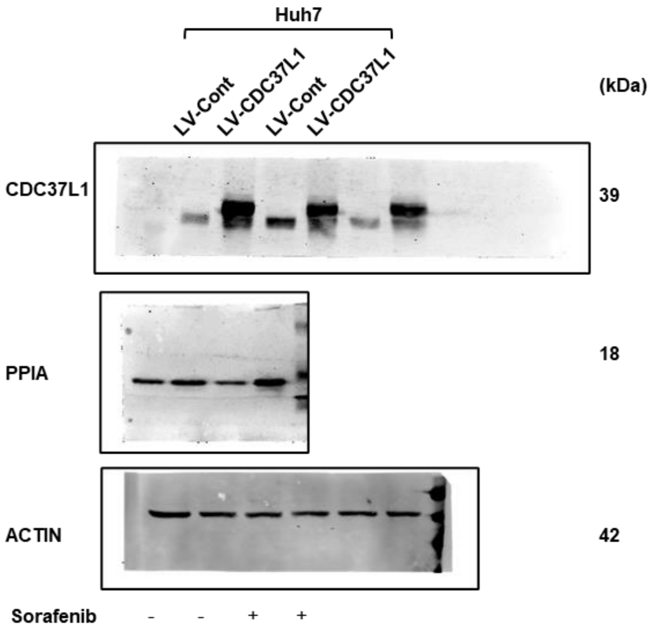
Huh7
LV-Cont
LV-CDC37L1
LV-Cont
LV-CDC37L1
(kDa)
CDC37L1
39
PPIA
18
ACTIN
42
Sorafenib
-
-
+
+

c

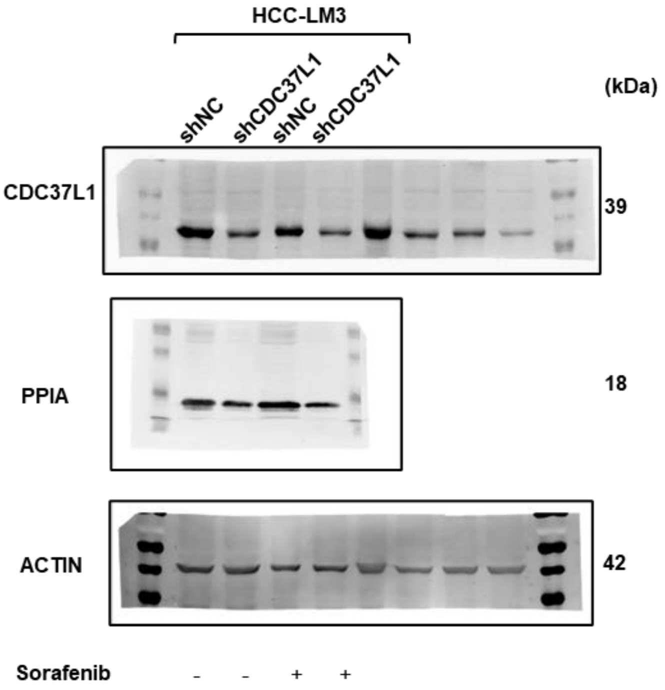
HCC-LM3
shNC
shCDC37L1
shNC
shCDC37L1
(kDa)
CDC37L1
39
PPIA
18
ACTIN
42
Sorafenib
-
-
+
+

e

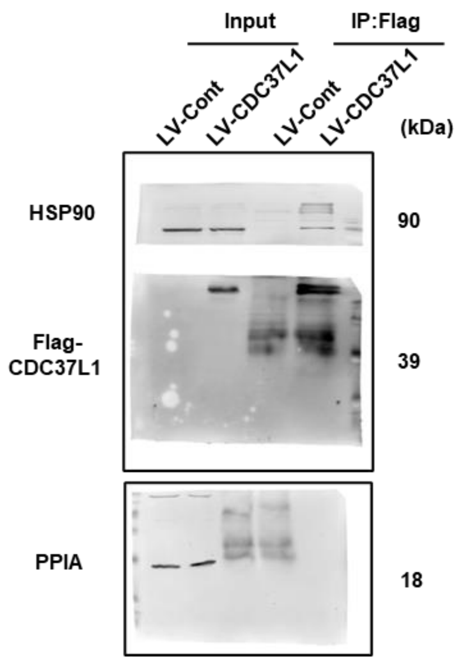
Input
IP:Flag
LV-Cont
LV-CDC37L1
LV-Cont
LV-CDC37L1
(kDa)
HSP90
90
Flag-
CDC37L1
39
PPIA
18

f

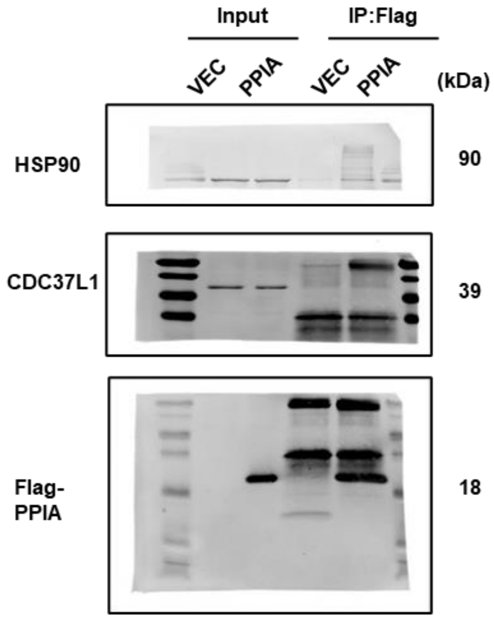
Input
IP:Flag
VEC
PPIA
VEC
PPIA
(kDa)
HSP90
90
CDC37L1
39
Flag-
PPIA
18

g

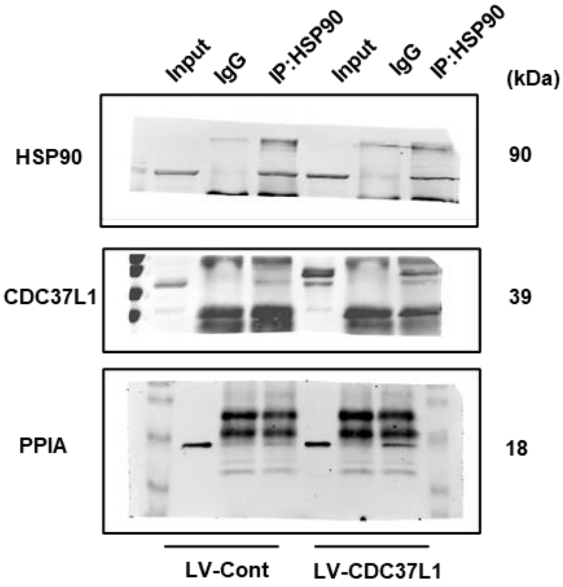
Input
IgG
IP:HSP90
Input
IgG
IP:HSP90
(kDa)
HSP90
90
CDC37L1
39
PPIA
18
LV-Cont
LV-CDC37L1
